# Mycosporine-like Amino Acids in *Palmaria palmata* (Rhodophyta): Specific Implication of Usujirene in Photoprotection

**DOI:** 10.3390/md22030121

**Published:** 2024-03-05

**Authors:** Fanny Lalegerie, Valérie Stiger-Pouvreau, Solène Connan

**Affiliations:** Univ Brest, CNRS, IRD, Ifremer, LEMAR, IUEM, F-29280 Plouzane, France; fannylalegerie@outlook.fr (F.L.); valerie.stiger@univ-brest.fr (V.S.-P.)

**Keywords:** MAAs, macroalga, nutrients, *Palmaria palmata*, photoprotection, pigments, UV radiation

## Abstract

The effect of UV radiation on the accumulation of mycosporine-like amino acids (MAAs) and pigments was investigated on red macroalga *Palmaria palmata* cultivated for 21 days. The data were combined with the effect of NaNO_3_ to further investigate the synthesis of these nitrogenous compounds. A progressive decrease in both total MAA and pigment contents was observed, with a positive effect of nitrate supply. Usujirene was the only MAA exhibiting a significantly increasing content when exposed to UV radiation, changing from 9% to 24% of the total MAA’s contribution, with no variation observed with NaNO_3_. This suggests a specific induction or synthesis pathway of usujirene for photoprotection, while the synthesis of other MAAs could have been limited by an insufficient amount of UV radiation and/or irradiance. The photoprotective ability of some MAAs could have been impacted by nitrogen starvation over time, resulting in a limited synthesis and/or potential use of MAAs as a nitrogen source for red macroalgae. The data confirmed the multiple effects of environmental factors on the synthesis of MAAs while providing new insights into the specific synthesis of usujirene, which could find an application in the cosmetics sector as natural sunscreen or an anti-ageing agent.

## 1. Introduction

Mycosporine-like amino acids (MAAs) are a group of secondary metabolites composed of an aminocyclohexenone or aminocyclohexeniminone ring, with a maximal absorption between 310 and 360 nm. They are present in many organisms such as bacteria, fungi, algae and animals such as squids [1,2,3]. More than 70 mycosporines and mycosporine-like amino acids have been characterized until now [2]. Due to their absorbance in both UV-A and UV-B wavelengths, they are generally described as UV-screening substances with photoprotective properties [1,4]. They are able to dissipate the absorbed energy and to prevent the formation of reactive oxygen species (ROS) [5]. In this way, many studies on red macroalgae have demonstrated a synthesis and accumulation of MAAs under UV exposition and/or high irradiance, coupled with a decrease in photosynthetic pigments. For example, 29% and 5% increases in MAAs in *Pyropia columbina*, exposed to UV-A and UV-B, respectively, have been observed [6]. Similarly, a correlation between palythinol amount and irradiance has been identified in *Gracilariopsis tenuifrons* [7]. More recently, 30%, 40% and 50% increases in total MAA content have been detected in *Halopithys incurva*, *Gracilariopsis longissima* and *Crassiphycus corneus,* respectively, when exposed to photosynthetically active radiation (PAR) + UV-A + UV-B treatment [8]. Moreover, the induction of the synthesis of shinorine, palythine and palythene in *Chondrus crispus* has been noticed when exposed for 24 h to PAR [9]. In the same way, it has been demonstrated that the synthesis of asterina-330, in wild/in situ populations of *Palmaria palmata*, is induced under high irradiance [10]. Due to their photoprotective role and high sun protection factor (SPF), MAAs are used as active ingredients in cosmetic products, such as HelioguardTM 365 (Mibelle Biochemistry, Buchs, Switzerland), or Helionori^®^ (Gelyma, Marseille, France), two natural sunscreens formulated with porphyra-334 extracted from red macroalga *Porphyra umbilicalis* to protect the skin against photo-ageing [11,12,13]. In addition to the photoprotective role, MAAs could have other bioactivities relevant for cosmeceutical applications [14], such as antioxidant [15,16], anti-inflammatory [17], or anti-collagenase activity [18], but could also exhibit anti-proliferative [19] and anti-cancer activities [5]. It has been shown, for example, that MAAs enhance collagen and elastin synthesis, contributing to the slowing down of skin ageing [20,21].

Although their photoprotective role is widely accepted, the role of each individual MAA is not completely understood. In addition, despite the genetic basis of MAAs biosynthesis having been mainly elucidated in cyanobacteria [22,23,24,25], the regulation of the synthesis pathway in red macroalgae is not completely elucidated [4,5,26]. MAAs constitute a diversified group [1], and some of them could have secondary roles (i.e., osmoregulation, thermoregulation [4]). The synthesis and accumulation of some MAAs could be induced under specific environmental conditions, with several studies suggesting that MAAs are not only dependent on light but also of other environmental parameters such as salinity or temperature [27]. Then, MAAs exhibit seasonal variations depending on each algal species [10,28,29]. More particularly, MAAs are nitrogenous compounds, and their synthesis could be affected by nutrient availability, as it has been suggested in *Porphyra umbilicalis* and *Neopyropia leucosticta* [30], or *Grateloupia lanceola* [31], whose MAA content appeared to depend on the ammonium level in the seawater. Similarly, it has been demonstrated in *Asparagopsis armata* that the content of individual MAAs was dependent on the nitrogen (N) level and that the shinorine percentage decreased with N fluxes compared to palythine, which increased [32]. More recently, in a previous one-year field study, we demonstrated that the accumulation of MAAs in *Palmaria palmata* under high irradiance could be limited by low-nutrient content in seawater in summer, or that some MAAs could be used as a nitrogen source [10]. On the contrary, the data reported the occurrence of asterina-330 only between March and July, suggesting a different synthesis pathway or a specific importance of this MAA in photoprotection [10].

In order to contribute to the general understanding of the synthesis of those compounds and highlight the specific role of some MAAs, the MAA and pigment accumulation under combined exposition to UV radiation and nutrient supply was investigated in the red macroalga *P. palmata*, known for its high diversity in MAAs [33]. Results could be used to orientate the metabolism towards the specific synthesis of some MAAs whose photoprotective role could be valorized in cosmetics.

## 2. Results

### 2.1. MAA Composition

**Figure 1:**
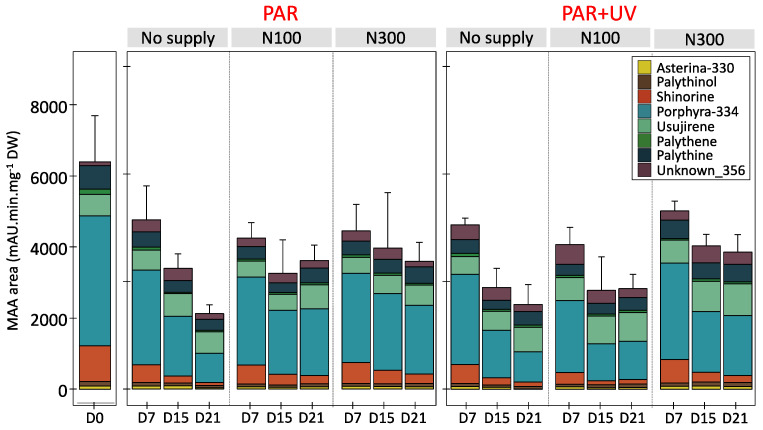
MAA areas (mAU min mg^−1^ DW) measured in *Palmaria palmata* during cultivation experiment, on 1^st^, 7^th^, 15^th^ and 21^st^ days (i.e., D0, D7, D15 and D21), under PAR or PAR + UV light, according to three nitrate levels (i.e., NaNO_3_ at 0 (seawater with no supply), 100 (N100) or 300 µM (N300)).

In all samples, whatever the duration or cultivation conditions, eight MAAs were identified in *Palmaria palmata*: shinorine, palythine, asterina-330, porphyra-334, palythinol, usujirene, palythene and an unknown MAA with a λ_max_ of 356 nm (described as Unknown_356; see Appendix 2 of [23] for the spectrum corresponding to MAA_15) (Figure 1). Porphyra-334 was the main MAA representing an average 49.51 ± 9.19% of the total MAA area (Table 1). The total MAA area decreased significantly with cultivation time, whatever the culture condition (Figure 1, Table S1). This temporal variation occurred mainly within the first week (post hoc Tukey HSD test, *p*-value < 0.001), with the total MAA area ranging on average from 6408.04 ± 1300.55 mAU·min·mg^−1^ DW at D0 to 4522.51 ± 584.78 mAU.min.mg^−1^ DW at D7. The decrease was smaller between D7 and D15, with ultimately no significant variation between the last two sampling points (D15 and D21), except in seawater with no nitrate supply under PAR conditions (Student’s test, *p*-value = 0.016).

Considering only the data from D7 to D21, shinorine, porphyra-334 and asterina-330 exhibited the most significant decrease (multi-way ANOVA, *p*-value < 0.001 for the 3 MAAs, for the factor ‘time’), while no significant temporal variations were observed for palythine and palythene (multi-way ANOVA, *p*-value = 0.16 and 0.16, respectively, for the factor ‘time’). Although there were no significant temporal variations (Table S1), usujirene was the only MAA for which the content tended to increase, especially under UV and high nitrate levels (N300), with an average 893.90 ± 92.63 mAU.min.mg^−1^ DW at D21, compared to 602.39 ± 186.74 mAU.min.mg^−1^ DW at D0. The contribution of usujirene to the total MAA content has, therefore, doubled during cultivation time, increasing from 9.29 ± 1.59% of the total MAA content at D0 to 23.89 ± 8.22% at D21 (multi-way ANOVA on % of usujirene from D0 to D21, *p*-value < 0.001, *p*-value < 0.001 and *p*-value = 0.13 for the factors ‘time, ‘light’ and ‘nutrient’, respectively; Table 1 and Table S1). Finally, the global proportion of palythinol varied from 1.95 ± 0.31% at D0 to 2.83 ± 0.57% at D21 (Table 1).

**Figure 2:**
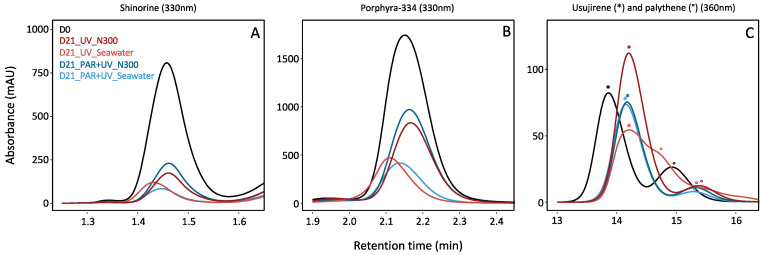
Zoom of HPLC chromatograms obtained from MAAs extracted from *Palmaria palmata*, after the 1^st^ and the 21^st^ days (i.e., D0 and D21) of cultivation. Colours represent the different light (i.e., PAR or PAR + UV) and nutrient conditions (i.e., NaNO_3_ at 0 (seawater with no supply), or 300 µM (N300)) (the average of the replicates was used to produce chromatograms). (**A**) Shinorine at 330 nm; (**B**) porphyra-334 at 330 nm; (**C**) usujirene (*) and palythene (°) at 360 nm (the average of the replicates was used to produce the figure, explaining slight shifts in retention times and the overlapping of usujirene and palythene at D21, under UV and without nitrate supply).

The addition of nitrate in the culture medium resulted in a positive effect on total MAA content over the cultivation period (Figure 1, Table S1). In seawater without nitrate supply, the decline of the total MAA area after 21 days reached 64% of the initial value, compared to only 42% in the 300 µM nitrate supply condition. However, not all MAAs were impacted in the same way, and three groups have been highlighted (Figure 2). First, shinorine, palythine and asterina-330 were highly impacted by nutrient levels, with no light effect (Table S1). The shinorine content at D21 was on average 106.02 ± 35.93, 179.57 ± 83.48 and 230.40 ± 73.39 mAU·min·mg^−1^ DW with no supply, or with the addition of 100 and 300 µM of nitrate, respectively. Then, porphyra-334 was also highly impacted by nitrate levels. However, a slightly higher content of porphyra-334 was also noticed under PAR radiations, especially when exposed to intermediate nitrate levels (Table 1, Figure 2). Finally, usujirene, palythene, palythinol and the unknown_356 levels showed no significant differences between different nitrate supplies, despite a tendency to increase with nitrate supply too (Figure 2, Table S1).

**Figure 3:**
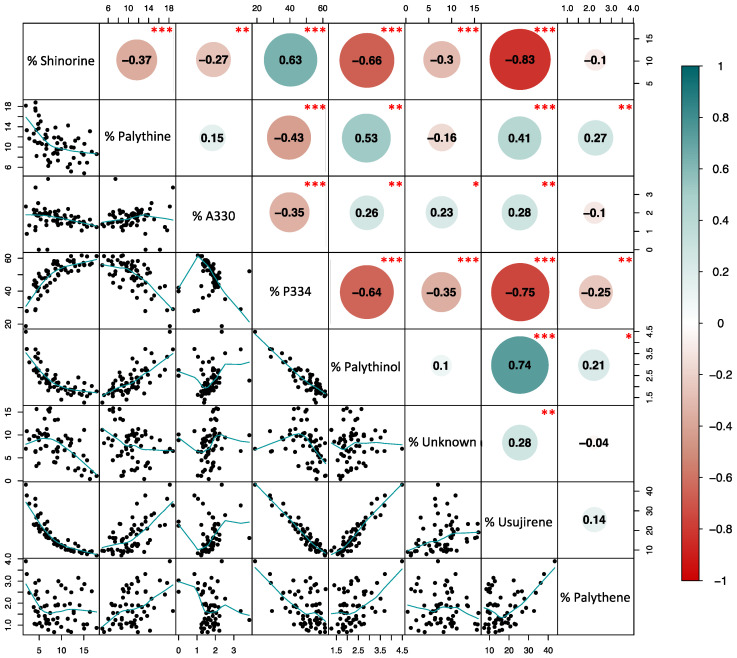
Matrix correlation between the proportion (%) of the different MAAs of *Palmaria palmata* cultivated for 21 days under different light and nutrient conditions. The values of the Kendall’s correlations (expressed inside green (positive) or red (negative) circles) are represented on the top of the diagonal. Stars represent the significant levels at 0.1% (***), 1% (**) and 5% (*). The corresponding bivariate scatter plots (with % MAAs in X-Y axis and a fitted line displayed for each relation) are on the bottom of the diagonal. The percent of each MAA was calculated related to the total MAA area (in mAU min mg^−1^ DW).

Usujirene and porphyra-334 were the only MAAs exhibiting a significant difference between PAR and PAR + UV (Table S1). Usujirene was the only one that exhibited a slightly higher content under PAR + UV than PAR, with an average of 797.47 ± 245.72 and 614.71 ± 185.44 mAU min mg^−1^ DW at D21, respectively. Although not significant, palythene, palythinol and the unknown_356 contents also tended to be higher under UV light (Table 1). In particular, the proportion of usujirene was highly positively correlated to palythinol (Kendall’s correlation τ = −0.74, *p*-value < 0.001; Figure 3). The content of asterina-330 tended also to increase under UV radiations, but only with a high nitrate supply (Student’s test, *p*-value = 0.010; Table S1, interaction N × L). The opposite pattern was observed for porphyra-334, whose proportions were negatively correlated with the proportion of usujirene and positively correlated to shinorine (Figure 3). With a NaNO_3_ supply of 100 µM, the porphyra-334 content decreased from 2048.32 ± 536.11 to 1376.54 ± 591.30 mAU min mg^−1^ DW under PAR and PAR + UV radiation, respectively. Although not significant, shinorine also tended to be higher with PAR light, i.e., 342.23 ± 190.55 compared to 284.96 ± 196.20 mAU min mg^−1^ DW under PAR + UV radiation.

### 2.2. Pigment Composition

**Figure 4:**
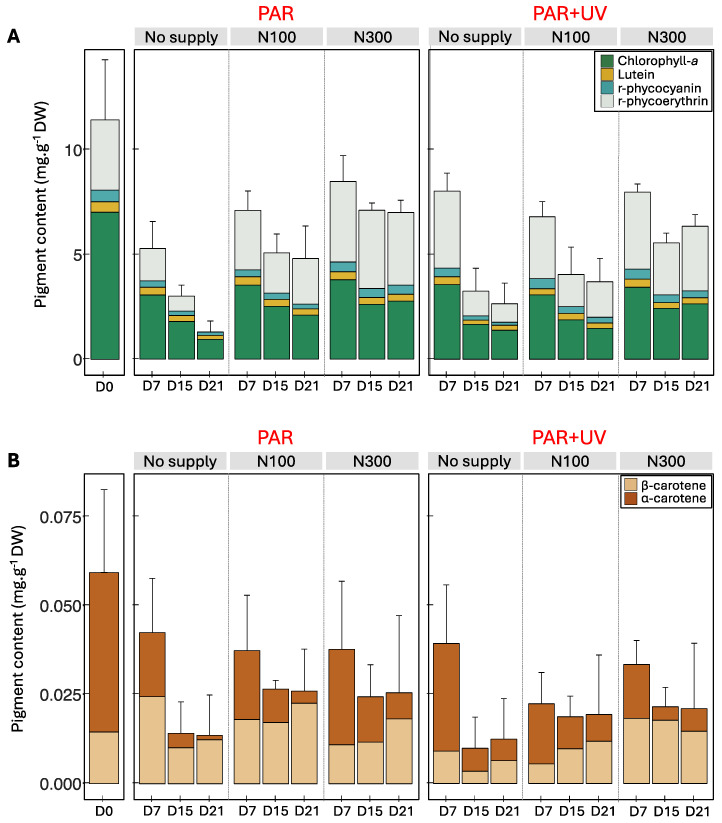
Pigment content (mg g^−1^ DW), i.e., (**A**) chlorophyll-*a*, lutein, r-phycoerythrin and r-phycocyanin, and (**B**) α-carotene and β-carotene measured in *Palmaria palmata* during cultivation experiment on 1^st^, 7^th^, 15^th^ and 21^st^ days (i.e., D0, D7, D15 and D21), under PAR or PAR + UV light according to three nitrate levels (i.e., NaNO_3_ at 0 (no supply), 100 (N100) or 300 µM (N300)).

Four apolar pigments have been identified in *P. palmata* by HPLC analysis, i.e., chlorophyll-*a*, lutein, α-carotene and β-carotene, in addition to the water-soluble pigments analysed by spectrophotometry, i.e., phycocyanin and phycoerythrin (Figure 4). The total pigment content declined between the initial date and the seventh day of cultivation under all conditions (Table S1). This decrease was due mainly to chlorophyll-*a*, which represented on average 87.27 ± 7.24% of the total liposoluble pigment content, varying from 7.03 ± 3.32 to 3.42 ± 0.45 mg g^−1^ DW during the first week (Figure 4A). A decrease over time was also observed for other pigments, except β-carotene for which the temporal variation was not significant due to a slightly higher content at D7 under PAR and no/low nutrient supply compared to the initial level (Figure 4, Table S1).

**Figure 5:**
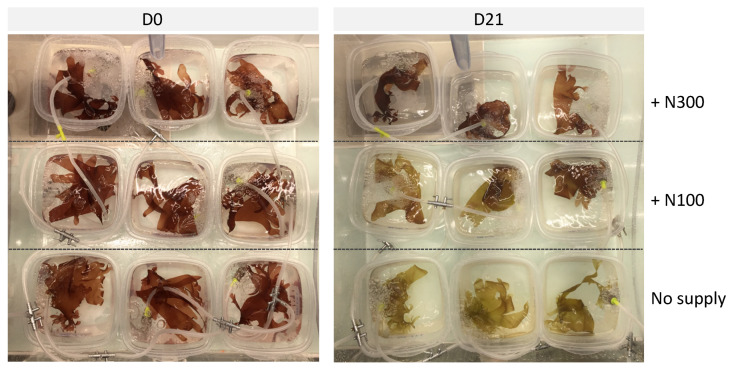
Cultivation tanks of *Palmaria palmata* at initial date (D0) and after 21 days (D21) of cultivation under PAR light conditions, exposed under three nitrate levels, i.e., NaNO_3_ at 0 (no supply), 100 (N100) or 300 µM (N300). Note the discolouration of thalli cultivated without N supply at D21.

The decrease in total pigment content continued after D7 (Figure 4), with a positive effect of the addition of nitrate on pigment levels, i.e., phycobiliproteins, chlorophyll-*a* and lutein (Table S1). Especially, nutrients resulted in maintaining an equivalent content over the cultivation period for phycoerythrin with 3.35 ± 1.25 mg g^−1^ DW at D0 and 3.28 ± 0.35 mg g^−1^ DW at D21 under a supply of 300 µM of NaNO_3_. Conversely, the phycoerythrin content declined in thalli cultivated in non-enriched seawater with an average of 0.44 ± 0.55 mg g^−1^ DW on D21, until becoming undetectable in some samples (Figure 4B). By consequence, without the addition of NaNO_3_, *P. palmata* thalli slowly changed from red to green after 21 days under cultivation (Figure 5). The chlorophyll-*a* content also significantly decreased from 7.03 ± 3.32 mg g^−1^ DW at D0 to 1.17 ± 0.55 mg g^−1^ DW at D21 in seawater with no supply compared to 2.71 ± 0.35 mg g^−1^ DW at D21 with a supply of 300 µM of NaNO_3_ (Table S1).

No significant difference was observed between PAR and PAR + UV on the total pigment content (two-way ANOVA on samples from D7 to D21, *p*-value = 0.836 for the factor ‘light’, *p*-value < 0.001 for the factor ‘nutrient’ and *p*-value = 0.085 for the interaction). β-carotene and lutein were the only pigments for which the concentration was slightly higher under PAR light (Table S1). The β-carotene content varied from 0.16 ± 0.00 mg g^−1^ DW to 0.01 ± 0.01 mg g^−1^ DW under PAR and PAR + UV radiations. The addition of nitrate resulted in the increase of concentrations only under UV radiation for β-carotene (two-way ANOVA on samples from D7 to D21, *p*-value = 0.017 for the factor ‘light’, *p*-value = 0.275 for the factor ‘nutrient’ and *p*-value = 0.029 for the interaction).

## 3. Discussion

To protect themselves against varying exposure to solar irradiance and specific harmful effects of UV radiations, intertidal macroalgae have the ability to synthesise photoprotective molecules, such as photoprotective pigments or mycosporine-like amino acids (MAAs) [34]. In particular, red algae represent a complex group with a high diversity of photoprotective molecules [35]. They possess varying metabolic profiles resulting from different synthesis pathways, including MAAs for which the specific role and the synthesis conditions of each one are still unclear. All species do not produce the same quantity/diversity of molecules, and some species favour a basic composition in high quantity, while others choose to produce a wide range of compounds in smaller quantities [33,36]. In this sense, HPLC analysis revealed in the present study a high MAA diversity in *Palmaria palmata*, as already described in [10].

Considering that the MAA and pigment contents decreased during the experiment and that this temporal decrease occurred mainly during the first week of cultivation, *P. palmata* may require a longer acclimation period. This period generally did not exceed a few days under cultivation to avoid thallus degradation over time. The synthesis of both MAAs and pigments in *P. palmata* could then have been limited by the cultivation conditions. An insufficient amount of PAR light may have been insufficient to induce the accumulation of photoprotective MAAs as the daily irradiance doses received by the cultured algae were quite low compared to solar radiation [37] and the ratio PAR/UV radiation was also much lower under laboratory conditions compared to natural solar light since the PAR light source cannot optimally simulate the natural light. This was already demonstrated in wild populations of *P. palmata*, with asterina-330, which is only synthesized during periods of high irradiance [10]. More specifically, MAA content may have declined due to the lack of specific UV-B radiations, as the importance of UV-A and UV-B together has already been demonstrated in MAA regulation [38] and the need for UV-B for the synthesis of some specific MAAs [39,40]. Chandra et al. also showed a 75% increase in the MAAs accumulation under UVB exposure over UVA exposure, and nearly 4-fold more than with PAR [41]. Figueroa et al. [37] even observed a 16-fold higher content of MAAs in the red macroalga *Gracilaria cornea* cultivated under solar radiation for 35 days. Considering pigments, the photosynthetic activity may have also been modified by the irradiance (PAR and PAR + UV) in the cultivation room (i.e., 65 µmol photons m^−2^ s^−1^, maximum limit of the laboratory equipment), which corresponds to low-light conditions commonly used in laboratory experiments (i.e., usually between 40 and 300 μmol photons m^−2^ s^−1^) [6,38,42,43,44]. However, samples were collected in March, corresponding to a winter phenotype, when individuals are already acclimated to relatively low irradiance. In addition, the level of photosynthetic chlorophyll-*a* and phycobiliproteins decreased over time whatever the condition, suggesting interaction with other parameters responsible for the general decrease in both MAAs and pigment content. In the case of summer phenotypes (e.g., samples collected in July), we could have expected to have individuals already acclimated to high irradiance, with a lower basal chlorophyll content (as observed in [10]; see low pigment content in *P. palmata* from May to August in Figure 3), for which we would not have observed a drop under cultivation condition. The harvesting period of the algae is thus of importance as the season and/or the year may impact the MAA content and composition [45]. The decrease observed during the present study may also have been caused by the difference in temperature between the cultivation room and in situ conditions at the collection time, i.e., 15–18 °C measured in the tanks, compared to not more than the 11 °C measured in situ in March. Some studies have already shown a negative effect of temperature on chlorophyll-*a* and MAA content [28], which could be related to a modification of the chemical kinetics and a slowing down of the photosynthetic metabolism [46].

Beyond light and temperature, the most likely hypothesis is that the algae suffered from a nitrogen deficiency under cultivation conditions that impacted the entire algal metabolism, including photosynthesis. Indeed, a significant effect of the nutrient concentrations in the culture medium was observed on the total pigment content (mainly due to a decrease in chlorophyll-*a* and phycobiliprotein contents) and total MAA area. As a consequence, *P. palmata* has increasingly lost its red colour during the cultivation experiment. At the end of our experiment, samples were completely yellow in seawater, as the lack of nutrient supply did not permit us to maintain the initial pigment concentration (Figure 4A; discolouration as illustrated in Figure 5). By contrast, samples remained red-coloured when supplied with 300 µM of NaNO_3_, due to the high level of phycoerythrin, the pigment characteristic of the colour of red macroalgae [47]. Similarly to the present study, in a 35-day outdoor cultivation experiment involving the effect of UV and nutrients, the phycobiliprotein content of *Gracilaria cornea* increased following the nitrogen addition but no effect of UV radiation was observed [37]. Data also showed that the levels of porphyra-334, shinorine and asterina-330 decreased during cultivation, but that the addition of nitrate resulted in a less significant decrease in MAA levels. Thus, the experiment highlighted a very clear positive effect of nitrate content on both MAAs and pigment contents, as suggested by previous studies on red seaweeds [6,30,48,49] including *P. palmata* [44].

The content of photosynthetic pigments decreases with low nutrient levels since photosynthetic activity is limited by Calvin’s cycle [48]. Then, the effect of nitrate can be explained by the fact that MAAs and most pigments contain nitrogen in their chemical structures, a starvation leading to a limited synthesis of these compounds. Such limitation could then explain the high decline observed within the first week of cultivation, which may be due to limited nutrient content in the tank compared to in situ conditions. In this way, results from the present cultivation experiment are in accordance with our previous in situ study, highlighting the relationship between MAA content and nutrient variations measured on wild/in situ populations of *P. palmata* [10]. The accumulation of those photoprotective compounds could be reduced in summer despite high light exposure due to a synthesis limited by the nutrient content in seawater with no supply. However, in order to determine if MAAs could act as nitrogen sources and storage and not only as photoprotectors as suggested in [43] or [10], it would be necessary to repeat the experiment with much higher nitrate contents in order to look at a possible induction of the synthesis of these compounds (i.e., an increase compared to day 0). Then, the present results highlighted three groups of MAAs in *P. palmata*: (1) those strongly dependent on nutrient content, with no effect of UV radiation (e.g., shinorine and palythine); (2) those moderately impacted by the addition of nitrate and which showed a low response to UV radiation (e.g., porphyra-334) and (3) those which did not appear to be impacted by nutrients, and which showed a significant increase in their synthesis under UV radiation (e.g., usujirene and palythinol). It can be suggested that the synthesis of MAAs belonging to the first and second groups was limited by nutrient content and that this indirectly limited their potential photoprotective role. For example, contrary to this study, it was observed that the content of shinorine and palythine increased in the red macroalga *Chondrus crispus* under UV-A radiation [40]; the low nitrate concentration in the present study could explain why we did not observe such a result.

A limitation in the synthesis of photoprotective compounds, due to an insufficient nutrient supply or UV light, could explain the absence of difference between PAR and PAR + UV radiations. In particular, palythinol did not exhibit a significantly higher content level under UV radiations while its content is well correlated to usujirene, as already demonstrated previously [10,50]. Some differences were expected as many studies show an impact of UV on the content of MAAs or photoprotective pigments of red macroalgae [6,40,43], including *P. palmata* [39]. Karsten et al. [9] showed, for example, that shinorine accumulation in *Chondrus crispus* is stimulated by UVR. However, other studies showed no effect of increased irradiance or UV radiation on red alga [51] or cyanobacteria [52]. In the case where UV addition does not result in changes in MAA content, or for species that did not produce MAAs or only a very small amount [33], it suggests the production of other photoprotective compounds such as phenolic compounds or physiological acclimation that allows them to withstand the increased UV radiation. Moreover, studies on the same cyanobacteria species did or did not have evidence of the effect of UV on MAA content, depending on phenotypes [52,53]. Thus, according to the species or the location/time of the collected sample, the content of some MAAs may change or not, linked to the modified irradiance. This variation may be linked to the pool of photoprotection and/or antioxidant molecules that these species produce and can easily and quickly be activated when needed. In our study, only usujirene exhibited a higher content under PAR + UV, suggesting a different behaviour of this MAA, which could have a different synthesis pathway less limited by nutrient content, or a specific role in photoprotection. In an in situ monitoring of MAA content in *P. palmata* [10], the content of asterina-330 increased in summer when irradiance was high compared to winter; the difference with the present study might be due to the irradiance as asterina-330 synthesis is favoured under UV-B and not UV-A radiation as observed in *C. crispus* [40]. The UV-protection role of usujirene has also been suggested by Yuan et al. [19], who identified usujirene only in the UV-exposed population of *P. palmata* compared to the more UV-protected population. Moreover, Conde et al. [54] indicated a different photoreactivity of cis-usujirene than its isomer trans-palythene, but both exhibited a strong photostability [55].

Contrary to porphyra-334 or shinorine, the level of usujirene did not vary with the different levels of nitrate supply, resulting in a higher relative proportion with time due to the decrease in the concentration of the main MAAs. Recently, Navarro et al. [56] also showed that the proportion of mycosporine-glycine in the red alga *Mazzaella laminarioides* does not change in response to nutrient supply, similarly to usujirene in the present study. These authors highlighted then the positive and negative impact of NO_3_^-^ on the proportion of shinorine and palythine. Data here suggest a different reactivity of usujirene in *P. palmata*, which may not serve as a source of nitrogen unlike other MAAs but as a UV-protective compound. In particular, the usujirene content was higher at D21 compared to D0; while the porphyra-334 content decreased over time, both MAAs being negatively correlated (Figure 3). This suggest either a specific induction pathway of usujirene such as a ‘metabolic pathway drift’ [26], or the transformation of porphyra-334 into usujirene, as in the theoretical synthesis pathway in which usujirene synthesis is derived from porphyra-334 [1,10,50,57]. This confirms that each MAA can have its own response, according to the considered species and environmental conditions. As observed for *C. crispus* [40], the theoretical synthesis pathways of main MAAs [50,57] and the results of the present study, the enzyme responsible for the synthesis of each MAA might be induced by either UV-B (asterina-330, palythinol, palythene; [40]), UV-A (shinorine and palythine [40]; usijirene and palythinol [the present study]); however, for shinorine and palythine the nitrogen concentration has apparently a higher effect than UVA on their synthesis. Moreover, as *P. palmata* could adjust its MAA content according to environmental conditions (here, UV light). It belongs to the Group 2 “species with a basic MAA concentration which is adjusted relative to changes in environmental radiation” defined by Hoyer et al. [36].

To conclude, the cosmetic industry is currently looking for a natural UV filter such as phenolic compounds or MAAs. MAAs arse real UV-screening molecules that absorb excessive energy from UV radiation and liberate heat without fluorescence nor radical production [54]. Moreover, Athukorala et al. [58] observed higher antioxidant activity of usujirene-enriched extracts compared to other porphyra-334-enriched extracts which could be explained by differences in their chemical structure [59]. Some solar products are already sold but only MAAs-enriched extracts from *Porphyra umbilicalis* (HelioguardTM365–Mibelle Group, Buchs, Switzerland; Helionori^®^–Gelyma, Marseille, France), *Polysiphonia elongata* (Ronacare^®^ RenouMer–Merck KGaA, Darmstadt, Germany) and *Gelidium corneum* (Alga-Gorria^®^–Laboratoire Biarritz, Biarritz, France) are commercially available [14,60]. By showing the specific anti-UV photoprotection capacity of usujirene compared to porphyra-334 or shinorine, for example, this study shows the potential use of *Palmaria Palmaria palmata* to produce extracts enriched in certain anti-UVA MAAs, which could participate in the diversification of exploited species/MAAs. In this regard, previous studies have demonstrated the high photostability of usujirene and the similar photoprotection ability of *P. palmata* extract to purified MAAs [55]. *Palmaria palmata* is naturally present and harvested on the European and American North Atlantic coasts [61] but also produced by aquaculture [61,62] and its metabolism could, therefore, be directed towards the synthesis of usujirene by modifying its culture condition (metabolic re-orientation) through for example the use of UV light.

## 4. Materials and Methods

### 4.1. Algal Collection and Cultivation Conditions

*Palmaria palmata* (Linnaeus) F. Weber & D. Mohr was collected in March at Portsall (Brittany, France, 48°33′53″ N—4°42′5″ W), at low tide. Thalli were cleaned in the laboratory with natural seawater, before being acclimated for 3 days under photosynthetically active radiation (PAR, 400–700 nm), with an intensity of 42.6 µmol photons m^−2^ s^−1^, and a 16 h light: 8 h dark cycle. After this period, samples were exposed either to PAR or PAR + UV radiation (addition of UVA 315–400 nm for a total intensity of 63.4 µmol photons m^−2^ s^−1^). The PAR radiation was provided by neon bulbs, while the PAR + UV radiation was provided by a DMX LED lighting ramp (Nicolaudie) associated with ESA Pro 2 software. In order to see only the effect of the UV radiation, the PAR provided by the neon bulbs has been reproduced as best as possible on the LED ramp. The daily irradiance dose was 7.7 µmol photons m^−2^ for the seaweeds under the PAR condition and 10.9 µmol photons m^−2^ of PAR and 196.9 kJ m^−2^ of UV for those under the PAR + UV condition. To test the effect of nutrients, samples (i.e., entire thalli) were supplied either with 0, 100 or 300 µM of NaNO_3_ for 21 days. The nutrient levels tested were determined according to previously published cultivation experiments carried out on MAAs content of other red macroalgae [6,20,21,29]. Samples were collected on days 0, 7, 15 and 21 of cultivation (i.e., D0, D7, D15 and D21 respectively), before being freeze-dried for subsequent metabolomic analyses. Each 1-L tank was equipped with a bubbling system, and seawater was renewed every three days, with an average temperature of 16.5 °C. The seawater used during the cultivation experiment was locally pumped and supplied by the French Research Institute for Exploitation of the Sea (IFREMER, Brittany, France, 48°21′33″ N—4°33′31″ W).

### 4.2. Extraction and Identification of Mycosporine-like Amino Acids

The mycosporine-like amino acids (MAAs) were extracted and analysed according to the protocol already described by Lalegerie et al. [33]. Three successive extractions were performed at 45 °C for 2 h, from 20 mg dry weight (DW) of algal powder and 70% aqueous ethanol. MAAs were analysed by high-pressure liquid chromatography (HPLC) using a Dionex Ultimate 3000 HPLC system (Thermo Scientific, Les Ulis, France) equipped with Zorbax Eclipse XDB C18 column (5 μm, 4.6 × 250 mm; Agilent, Santa Clara, CA, USA) and diode-array detector (DAD). Each sample was dissolved in 500 μL of 2.5% aqueous methanol with 0.1% acetic acid (diluted in MilliQ water) and filtrated (0.2 μm) before injection of 20 μL. The HPLC parameters were as follows: isocratic mode with 0.1% acetic acid in Milli-Q water as mobile phase; flow rate of 1 mL min^−1^; column temperature of 25 ± 1 °C; run time of 20 min; detection at 320, 330, 334 and 360 nm. Due to the consistency of MAAs composition within a species, identification was done by comparison of absorption spectra and retention time of MAAs in *Palmaria palmata*, identified by LC-MS analysis in our previous study and using the same HPLC method [33]. As there is no commercial standard for MAAs, the peak area of each MAA was adjusted to the volume of extract and the quantity of algal powder used for extraction and results were thus expressed in mAU.min.mg^−1^ DW in order to compare the different treatments. HPLC data acquisition was done by Chromeleon 7 software (Thermo Scientific Dionex, Les Ulis, France).

### 4.3. Extraction and Quantification of Pigments

Chlorophyll-*a* and carotenoids were extracted using 75 mg DW mixed with 750 μL 90% aqueous acetone according to the protocol of Schmid and Stengel [63]. Apolar pigments were separated according to the protocol already described in Lalegerie et al. [33] using an HPLC Dionex Ultimate 3000 system equipped with a diode array detector (Thermo Scientific) and an ACE C18 column (150 × 4.6 mm, 3 μm). Identification and quantification of pigments were then determined using standard curves obtained from commercial standards (chlorophyll-*a* from Sigma (St. Louis, MO, USA), and α-carotene, β-carotene, lutein from DHI (Hørsholm, Denmark)). In addition to apolar pigments, phycobiliproteins (i.e., phycoerythrin and phycocyanin) were extracted from 75 mg DW of algal powder mixed with 1.5 mL of phosphate buffer (0.1 M, pH 6.8), according to the protocol of Sun et al. [64], already adapted in Lalegerie et al. [33]. Phycobiliproteins were quantified by spectrophotometry using the equations from Beer and Eshel [65].

### 4.4. Data Analysis

Statistical analyses were performed using R software (version 3.6.1) [66] through the integrated development environment Rstudio (version 1.2.5001) [67]. Several thalli (*n* = 3) were cultivated for each treatment (light × nutrients) and used as replicates; with results being expressed as mean ± standard deviation. At D0, the algae were exposed to a single condition, thus more replicates were processed (*n* = 6) to avoid a severely imbalanced data set when running statistical analysis. The effect of light and/or nutrients was analysed by Student’s test or multi-way ANOVA, according to the “time”, “light”, and/or “nutrient” factors. Tukey’s post hoc test was performed following ANOVA in order to compare the different groups. Correlations between MAAs and pigments were studied by calculating Pearson’s coefficients (or Kendall’s coefficients if the normality was not respected) with corrplot(x) and chart.Correlation(x) functions. The normality and homogeneity of variances were checked with the Shapiro test and F-test respectively. In order to see variations between each MAA in relation to synthesis pathway, the percentages of each MAA were calculated based on the total MAAs area.

## Figures and Tables

**Table 1:** Percent of each MAA in *Palmaria palmata*, according to days of cultivation, light (i.e., PAR or PAR + UV) and nutrient conditions (i.e., NaNO_3_ at 0 (seawater with no supply), 100 (N100) or 300 µM (N300)).

Days	Light	Nutrient	% Shinorine	% Palythine	% A330	% P334	% Palythinol	% Unknown	% Usujirene	% Palythene
D0	PAR	Seawater	15.73 + 1.53	10.34 + 1.81	1.41 + 0.27	57.38 + 3.57	1.95 + 0.31	1.63 + 0.89	9.29 + 1.59	2.28 + 0.86
D7	PAR	Seawater	10.49 + 1.51	8.97 + 2.10	1.75 + 0.24	56.22 + 2.02	2.00 + 0.15	7.10 + 0.53	11.82 + 1.37	1.65 + 0.50
		N100	12.53 + 3.31	8.49 + 1.49	1.49 + 0.22	58.24 + 2.52	1.78 + 0.26	5.59 + 0.98	10.54 + 3.51	1.33 + 0.30
		N300	13.28 + 1.45	8.77 + 1.08	1.62 + 0.19	56.09 + 3.46	1.84 + 0.03	6.67 + 2.06	10.04 + 0.44	1.69 + 0.74
	PAR+UV	Seawater	11.57 + 2.37	8.27 + 3.20	1.68 + 0.39	54.81 + 4.70	1.85 + 0.29	9.06 + 0.20	10.70 + 1.85	2.06 + 0.86
		N100	8.14 + 1.58	7.62 + 1.75	1.79 + 0.33	50.03 + 4.90	1.74 + 0.29	13.31 + 4.12	15.65 + 3.91	1.72 + 1.02
		N300	13.2 + 1.80	10.11 + 4.82	1.78 + 0.42	54.06 + 2.45	1.81 + 0.47	5.38 + 4.97	12.73 + 2.76	0.94 + 0.25
D15	PAR	Seawater	5.91 + 0.97	9.65 + 1.92	2.59 + 1.09	49.57 + 2.23	2.28 + 0.09	10.17 + 0.25	18.75 + 2.31	1.09 + 0.15
		N100	8.38 + 4.25	9.10 + 3.75	1.59 + 0.50	53.77 + 8.88	2.05 + 0.60	8.46 + 1.24	15.16 + 7.05	1.48 + 0.69
		N300	9.66 + 1.41	10.1 + 1.43	1.66 + 0.07	54.01 + 2.17	2.02 + 0.25	8.27 + 1.79	12.66 + 2.49	1.61 + 0.93
	PAR+UV	Seawater	7.22 + 1.71	9.11 + 2.22	1.98 + 0.23	46.86 + 4.15	2.23 + 0.35	12.42 + 1.83	18.46 + 4.54	1.73 + 0.27
		N100	4.43 + 2.23	11.16 + 2.63	1.65 + 0.69	37.89 + 8.68	2.87 + 0.75	12.62 + 2.66	27.30 + 9.24	2.08 + 1.16
		N300	6.65 + 2.24	11.56 + 6.34	2.43 + 0.82	41.81 + 11.10	2.73 + 0.84	11.63 + 4.53	21.54 + 9.71	1.65 + 0.59
D21	PAR	Seawater	4.10 + 1.28	14.64 + 2.79	1.40 + 0.22	38.78 + 3.78	3.00 + 0.20	7.46 + 1.43	28.41 + 5.41	2.22 + 0.37
		N100	6.43 + 1.99	11.63 + 1.88	1.67 + 0.18	52.06 + 6.59	2.32 + 0.45	5.69 + 3.52	18.76 + 6.99	1.43 + 0.98
		N300	7.52 + 2.28	12.77 + 1.73	1.74 + 0.24	54.12 + 3.76	2.46 + 0.32	4.30 + 0.69	15.62 + 4.65	1.47 + 0.40
	PAR+UV	Seawater	5.29 + 1.39	15.75 + 2.17	0.35 + 0.61	36.80 + 7.37	2.87 + 0.44	8.46 + 1.91	27.66 + 7.41	2.82 + 0.27
		N100	4.43 + 2.31	13.13 + 4.37	1.84 + 0.50	37.02 + 16.95	3.44 + 0.98	8.55 + 2.17	29.40 + 12.81	2.21 + 1.48
		N300	5.05 + 1.26	12.73 + 1.71	2.16 + 0.32	43.22 + 7.02	2.89 + 0.56	8.96 + 1.16	23.47 + 4.06	1.52 + 0.60

## Data Availability

The raw data supporting the conclusions of this article will be made available by the authors on request.

## Supplementary Materials

The following supporting information can be downloaded at https://www.mdpi.com/article/10.3390/md22030121/s1, Table S1: Results of the multi-way ANOVA testing the effect of cultivation conditions (i.e., time (T), nutrient level (N) and light (L)) on the mycosporine-like amino acid (MAAs, in mAU.min.mg^−1^ DW) and pigment (mg g^−1^ DW) compositions of *Palmaria palmata*.
