# Supplementary material for: Mycosporine-like Amino Acids in Palmaria palmata (Rhodophyta): Specific Implication of Usujirene in Photoprotection
Source: Mar Drugs. 2024 Mar 5;22(3):121. doi: 10.3390/md22030121 (PMC10972262; doi:10.3390/md22030121)
Supplement: Supplementary file 1 [file marinedrugs-22-00121-s001.zip › marinedrugs-2876636-supplementary Table S1.pdf]

	Variables		Factors: Time	Nutrients	Light	Interactions: T x N	T x L	N x L	T x N x L
MAAs	Shinorine	F	87.052	5.771	3.009	0.808	0.280	2.925	0.456
		p -value	**< 2.00e-16 *****	**0.006 ****	0.090	0.528	0.757	0.065	0.768
	Palythine	F	11.016	4.716	0.085	0.488	0.003	0.958	0.567
		p -value	**1.95e-05 *****	**0.0143 ***	0.772	0.7443	0.997	0.392	0.688
	Asterina-330	F	9.217	6.947	0.067	5.184	0.398	5.281	0.433
		p -value	**8.78e-05 *****	**0.003 ****	0.797	**0.002 ****	0.674	**0.009 ****	0.784
	Porphyra-334	F	46.312	5.324	6.559	2.309	0.796	1.834	0.231
		p -value	**3.13e-13 *****	**0.009 ****	**0.0142 ***	0.0741	0.458	0.173	0.920
	Palythinol	F	6.051	2.786	1.282	1.319	0.43	1.100	0.176
		p -value	**0.002 ****	0.073	0.264	0.279	0.653	0.343	0.949
	Unknown_356	F	10.788	0.041	10.732	1.656	0.148	0.928	2.128
		p -value	**2.35e-05 *****	0.960	**0.002 ****	0.179	0.863	0.403	0.095
	Usurijene	F	1.820	0.459	7.508	0.121	0.181	2.658	0.325
		p -value	0.159	0.635	**0.009 ****	0.974	0.835	0.082	0.859
	Palythene	F	12.418	0.157	0.426	0.803	0.099	0.526	0.111
		p -value	**6.47e-06 *****	0.855	0.518	0.530	0.906	0.595	0.978
	SUM MAAs	F	34.713	5.595	0.211	1.984	0.300	1.153	0.243
		p -value	**2.47e-11 *****	**0.007 ****	0.649	0.115	0.743	0.326	0.912
Pigments	Chlorophyll-*a*	F	48.333	14.180	1.732	2.511	0.284	3.296	0.300
		p -value	**6.36e-11*****	**2.87e-05*****	0.196	0.059	0.754	**0.049***	0.876
	Lutein	F	25.380	8.115	7.107	3.329	0.910	1.128	2.248
		p -value	**1.33e-07*****	**0.001****	**0.011***	**0.020***	0.412	0.335	0.083
	α-carotene	F	30.036	0.157	0.007	0.999	0.645	4.845	0.832
		p -value	**2.12e-08*****	0.855	0.933	0.421	0.531	0.014	0.514
	β-carotene	F	0.625	1.245	5.740	1.210	0.355	3.597	0.550
		p -value	0.541	0.300	**0.022***	0.324	0.704	0.038	0.700
	r -phycoerythrin	F	23.261	52.802	0.364	3.261	3.815	10.920	0.589
		p -value	**3.28e-07*****	**1.97e-11*****	0.550	**0.022***	**0.031***	**0.000*****	0.672
	r -phycocyanin	F	26.581	34.135	1.053	1.280	4.238	4.257	0.144
		p -value	**8.13e-08*****	**4.86e-09*****	0.312	0.296	**0.022***	**0.022***	0.965
	SUM pigments	F	55.535	52.649	0.154	4.412	2.656	9.308	0.489
		p -value	**9.95e-12*****	**2.05e-11*****	0.697	**0.005****	0.084	**0.001*****	0.744

Significant p-values are indicated in bold and stars according to the following levels:

* (p-value ≤ 0.05); ** (p-value ≤ 0.01); ** (p-value ≤ 0.001)
